# Data-driven discovery of a novel sepsis pre-shock state predicts impending septic shock in the ICU

**DOI:** 10.1038/s41598-019-42637-5

**Published:** 2019-04-16

**Authors:** Ran Liu, Joseph L. Greenstein, Stephen J. Granite, James C. Fackler, Melania M. Bembea, Sridevi V. Sarma, Raimond L. Winslow

**Affiliations:** 1 Institute for Computational Medicine, The Johns Hopkins University, Maryland, USA; 2 Department of Biomedical Engineering, The Johns Hopkins University School of Medicine & Whiting School of Engineering, Maryland, USA; 3 Department of Anesthesiology and Critical Care Medicine, The Johns Hopkins University School of Medicine, Maryland, USA

## Abstract

Septic shock is a life-threatening condition in which timely treatment substantially reduces mortality. Reliable identification of patients with sepsis who are at elevated risk of developing septic shock therefore has the potential to save lives by opening an early window of intervention. We hypothesize the existence of a novel clinical state of sepsis referred to as the “pre-shock” state, and that patients with sepsis who enter this state are highly likely to develop septic shock at some future time. We apply three different machine learning techniques to the electronic health record data of 15,930 patients in the MIMIC-III database to test this hypothesis. This novel paradigm yields improved performance in identifying patients with sepsis who will progress to septic shock, as defined by Sepsis- 3 criteria, with the best method achieving a 0.93 area under the receiver operating curve, 88% sensitivity, 84% specificity, and median early warning time of 7 hours. Additionally, we introduce the notion of patient-specific positive predictive value, assigning confidence to individual predictions, and achieving values as high as 91%. This study demonstrates that early prediction of impending septic shock, and thus early intervention, is possible many hours in advance.

## Introduction

Sepsis is a life-threatening organ dysfunction caused by a dysregulated host response to infection^1^. Septic shock is an advanced state of sepsis characterized by circulatory, cellular, and metabolic abnormalities that are associated with a greater risk of mortality than sepsis alone^1^. Sepsis and septic shock are the leading causes of hospital mortality, accounting for 37–56% of all inpatient deaths^2^. They have been the most costly medical conditions across all payers since 2008^3^. Septic shock is particularly lethal, with mortality estimated as high as 45%^4,5^. More than a decade ago, Kumar *et al*. found that septic shock patients treated within the first hour of diagnosis had a survival rate of 80%, every hour of delayed treatment increased mortality by ~8%^6^, and average delay between diagnosis and treatment was 6 hours^6^. More recent studies have corroborated this finding of increased mortality with delayed treatment^7–9^. Despite these findings, delayed treatment remains common in current practice^7–9^. Therefore, an automated system able to identify patients with sepsis who are likely to transition to septic shock well in advance of that transition has the potential to improve patient outcomes by providing an early time window for intervention.

A number of computational approaches to early prediction of sepsis and septic shock using Electronic Health Record (EHR) data have been developed. Recently, Henry *et al*.^10^ and Nemati *et al*.^11^ used Cox proportional hazards models for early prediction of sepsis and septic shock, respectively. Mao *et al*.^12^ applied gradient boosting to early prediction of both sepsis and septic shock. A limitation of the methods presented by Nemati *et al*.^11^ and Mao *et al*.^12^ is that because they make predictions at a fixed time interval prior to onset, this classification task is only feasible in retrospective studies when the time of sepsis onset or septic shock onset is known. Here, we develop a novel statistical learning approach for identifying sepsis patients who are at elevated risk of transitioning to septic shock, as diagnosed using the Third International Consensus Definitions for Sepsis and Septic Shock (Sepsis-3)^1^. This approach is suitable for prospective study and evaluation in real time. The method also provides a patient-specific positive predictive value (PPV; the probability the prediction is correct) each time a prediction of impending sepsis generates an alert.

The foundation of our approach is the assumption that there exists a clinical state of sepsis that we refer to as the “pre-shock” state. The existence of this pre-shock state is predicated on the fact that the physiology of sepsis patients who progress to septic shock must be changing gradually over time as their condition worsens, and therefore these patients will first transition into the pre-shock state before entering septic shock. Early prediction of those patients who will ultimately develop septic shock therefore corresponds to identifying those patients who enter the pre-shock state. To do this, we apply machine learning to features calculated from patient EHR data to estimate whether or not a patient enters this pre-shock state, and if they do, the time of transition. We demonstrate this novel paradigm using three different machine learning methods (generalized linear models (GLM), XGBoost^13^, recurrent neural networks^14^ (RNN)), yielding improved performance in early prediction of impending septic shock relative to existing methods, achieving a maximum area under the receiver operating characteristic curve (AUC) of 0.93, with 88% sensitivity, 84% specificity, overall 52% PPV, and a median early warning time (EWT) of 7 hours prior to septic shock onset with the best-performing method (RNN). We show that pre-shock is primarily characterized by elevated lactate, cardiovascular sequential organ failure assessment (SOFA) score^15^, heart rate, partial pressure of oxygen, fraction of inspired oxygen, and decreased Glasgow Coma Score (GCS)^16^.

## Results

**Figure 1:**
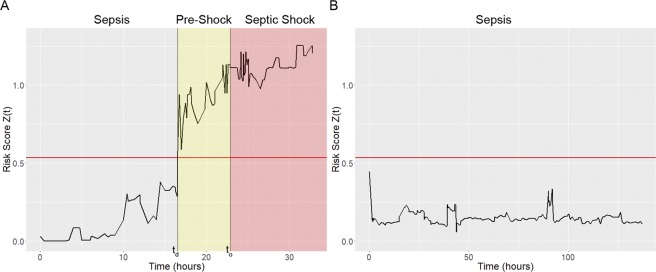
Risk score *z*(t) (black line) trajectories for a septic shock patient ((**A**) MIMIC-III subject 250) and a non-shock sepsis patient ((**B**) subject 21). The detection threshold θ applied to *z*(t) is indicated by the red horizontal line. In panel A, patient state transitions into the pre-shock state when the risk exceeds the threshold θ at detection time t_d_. Septic-shock is clinically diagnosed at onset time t_o_. Time is given in hours relative to the start of observations, and EWT in this example is ~6 hours.

A time-evolving risk score is generated by applying a chosen machine learning method (e.g. GLM, XGBoost, RNN) to EHR data to model the risk of future septic shock onset. This risk score is updated each time a new patient feature becomes available. The first time at which the risk score exceeds a fixed threshold (detection time t_d_) defines the transition into the pre-shock state and is the time at which we predict that it is highly likely a given patient will ultimately transition from sepsis to septic shock. Early warning time (EWT) is defined as the difference between shock onset time (t_o_) and detection time (t_d_). Figure 1 shows example risk score trajectories using XGBoost from a patient who does (Fig. 1A) and a patient who does not (Fig. 1B) progress from sepsis to septic shock.

**Figure 2:**
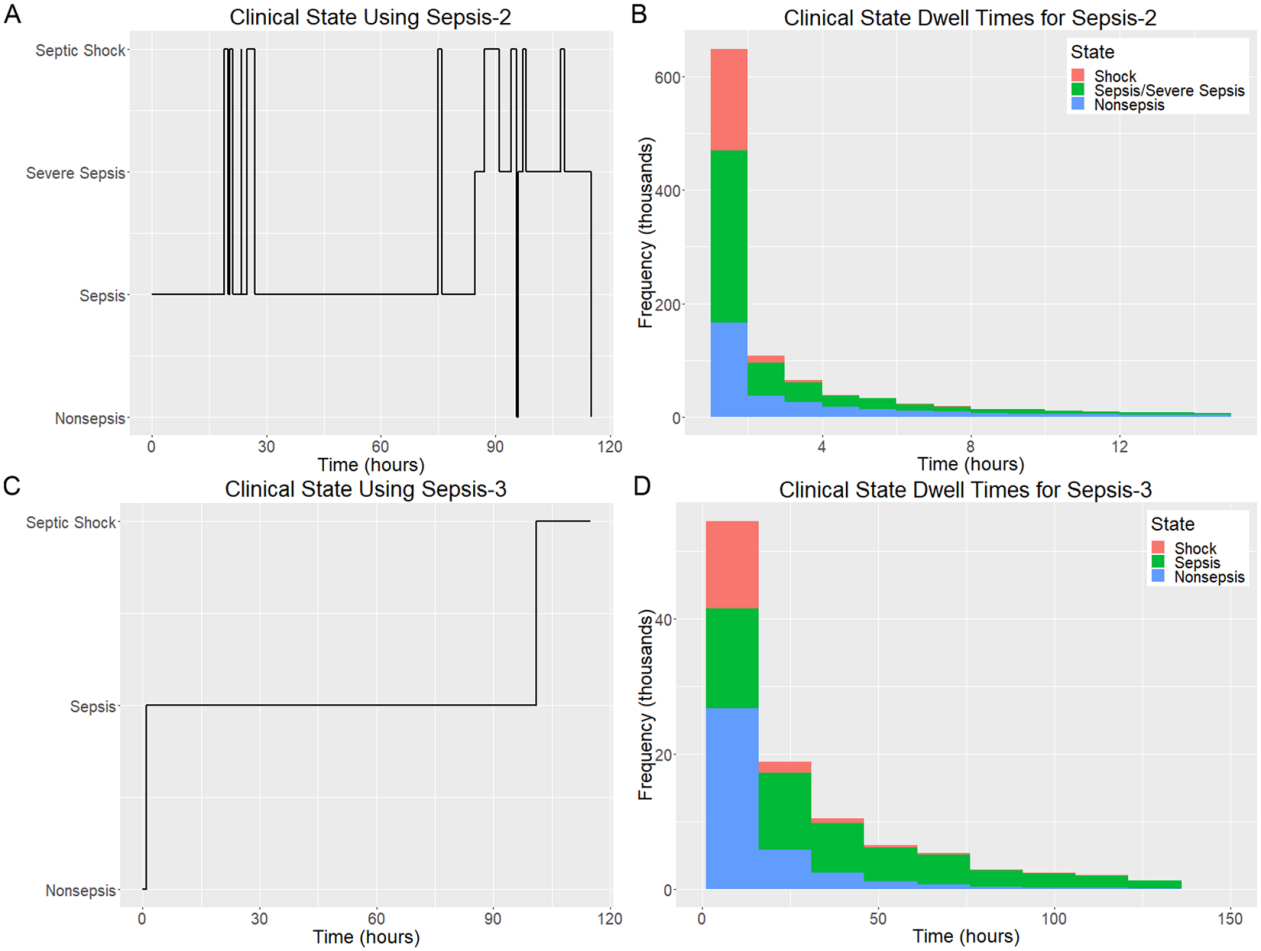
Comparison of Sepsis-2 and Sepsis-3 clinical state label characteristics calculated from EHR data in the study population. (**A**) Time evolution of Sepsis-2 labels for MIMIC-III subject 3205. (**B**) Sepsis-2 state dwell time distributions for non-sepsis, sepsis/severe sepsis, and septic shock. (**C**) Time evolution of Sepsis-3 labels for subject 3205 (**D**) Sepsis-3 state dwell time distributions for non-sepsis, sepsis, and septic shock.

Our method for prediction of impending transition into septic shock was applied to EHR data from the version 1.4 MIMIC-III database^17^ (see Fig. S4 for exclusion criteria). Clinical state labels were determined computationally by applying the Sepsis-3 definitions for Sepsis and Septic Shock^1^. In order to make comparisons to methods developed previously, clinical state labels were also determined using the Sepsis-2 definitions^18^. We found that the Sepsis-2 clinical state labels are highly unstable, fluctuating frequently over time, thus making it unclear exactly when a patient entered septic shock or any other clinical state. Figure 2A shows an example of clinical state labels generated using the Sepsis-2 criteria for a representative patient. To support more direct comparison with Sepsis-3, the sepsis and severe sepsis states as defined by Sepsis-2 criteria were combined into an aggregate state. Sepsis-2 clinical state dwell time distributions for the 15,930 patients included in this study (Fig. S4) are shown for non-sepsis, sepsis/severe sepsis, and septic shock in Fig. 2B. Mean dwell times in each clinical state are 6.3 hours, 6.1 hours, and 2.0 hours respectively. Figure 2C shows Sepsis-3-based clinical state labels for the same patient as in Fig. 2A. Figure 2D shows Sepsis-3 clinical state dwell time distributions for the same set of patients as in Fig. 2B for non-sepsis, sepsis, and septic shock, with mean dwell times of 24.9 hours, 73.1 hours, and 15.7 hours, respectively. The mean number of label changes per patient in this same group of patients is 17.2 with a median of 8 when using Sepsis-2 criteria, whereas the mean number of label changes is 1.5 with a median of 1 when using Sepsis-3 criteria. The Sepsis-2-based clinical labels are temporally unstable, unlike those based on the Sepsis-3 criteria. Clinical state labels for the methods described in this study therefore adhere to Sepsis-3 definitions.

**Figure 3:**
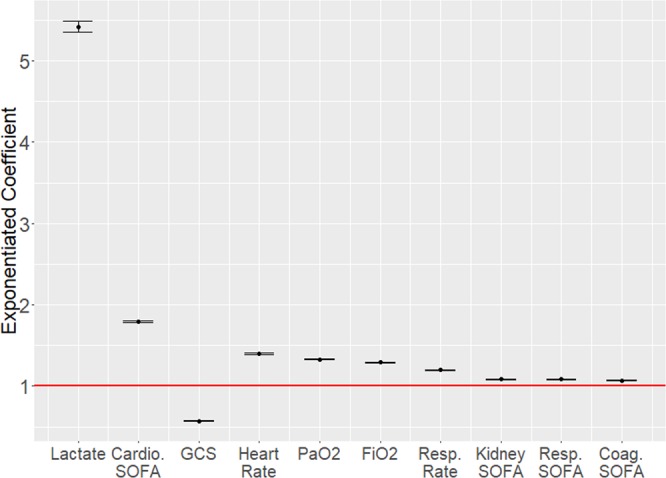
Exponentiated model coefficients and 95% confidence bounds for the ten selected features from one sample train/test iteration. These coefficients were learned using features normalized to a mean of 0 and unit standard deviation. Abbreviations: Cardio SOFA – Cardiovascular SOFA score; PaO2 – Partial pressure of oxygen; FiO2 – Fraction of inspired oxygen; Resp. Rate – Respiratory Rate; Resp. SOFA – Respiratory SOFA score; Coag. SOFA – Coagulatory SOFA score.

**Figure 4:**
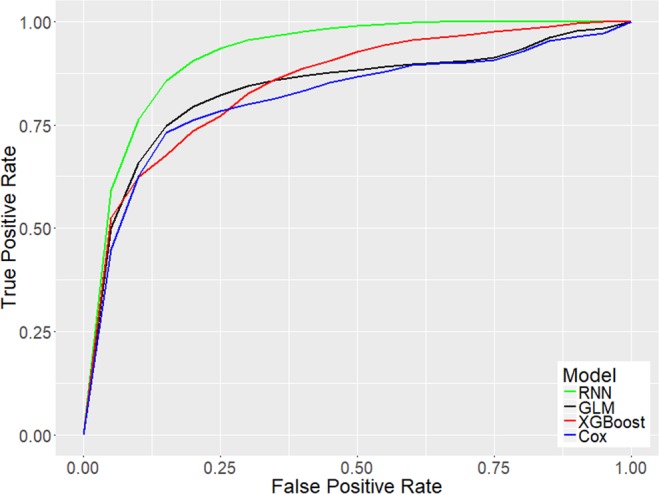
Mean ROC curves for detection methods with risk score computed using the pre-shock modeling approach presented here using GLM (black), XGBoost (red), or RNN (green) or a Cox hazard model as proposed previously (blue)^10^. Clinical state labels were determined using Sepsis-3 criteria.

GLM, XGBoost, and RNN were trained using data from two groups of sepsis patients: those in the first group never deteriorated into septic shock, whereas those in the second group did. Within this second group, EHR data over a time window immediately preceding the transition into septic shock were used (see Methods: *Risk Modeling*). Among the three methods used, GLM yields the most readily interpretable models. Figure 3 shows exponentiated coefficients of the GLM for the ten features identified as yielding the greatest detection performance, from left to right in descending order of relative importance. These coefficients indicate the impact of each feature on the risk score and can be interpreted as odds ratios. For example, the coefficient for lactate is approximately 5, therefore a patient with serum lactate concentration 1 standard deviation above the population mean is approximately five times as likely to transition into shock than a patient with average serum lactate concentration (see Discussion: *Interpretation of Model*). We also applied the Cox proportional hazards approach described by Henry *et al*.^10^ to the same data and compared results. Figure 4 shows the receiver operating characteristic (ROC) curves generated for early prediction using GLM, XGBoost, RNN, and the Cox proportional hazards model. Table 1 lists AUC, sensitivity, specificity, PPV, and median early warning time (EWT) for each approach.

**Table 1:** Performance metrics for each of the four evaluated strategies for early prediction of septic shock.

Method	AUC	Sensitivity	Specificity	PPV	Median EWT (hrs)
GLM	0.87*	0.82*	0.83	0.49	6.9*
XGBoost	0.85*	0.76	0.79	0.43	6.0
RNN	0.93*	0.88*	0.84	0.52*	7.0*
Cox	0.82	0.76	0.82	0.47	6.1

An asterisk (*) indicates that the performance metric is significantly higher (p < 0.01) than the corresponding metric for Cox.

**Figure 5:**
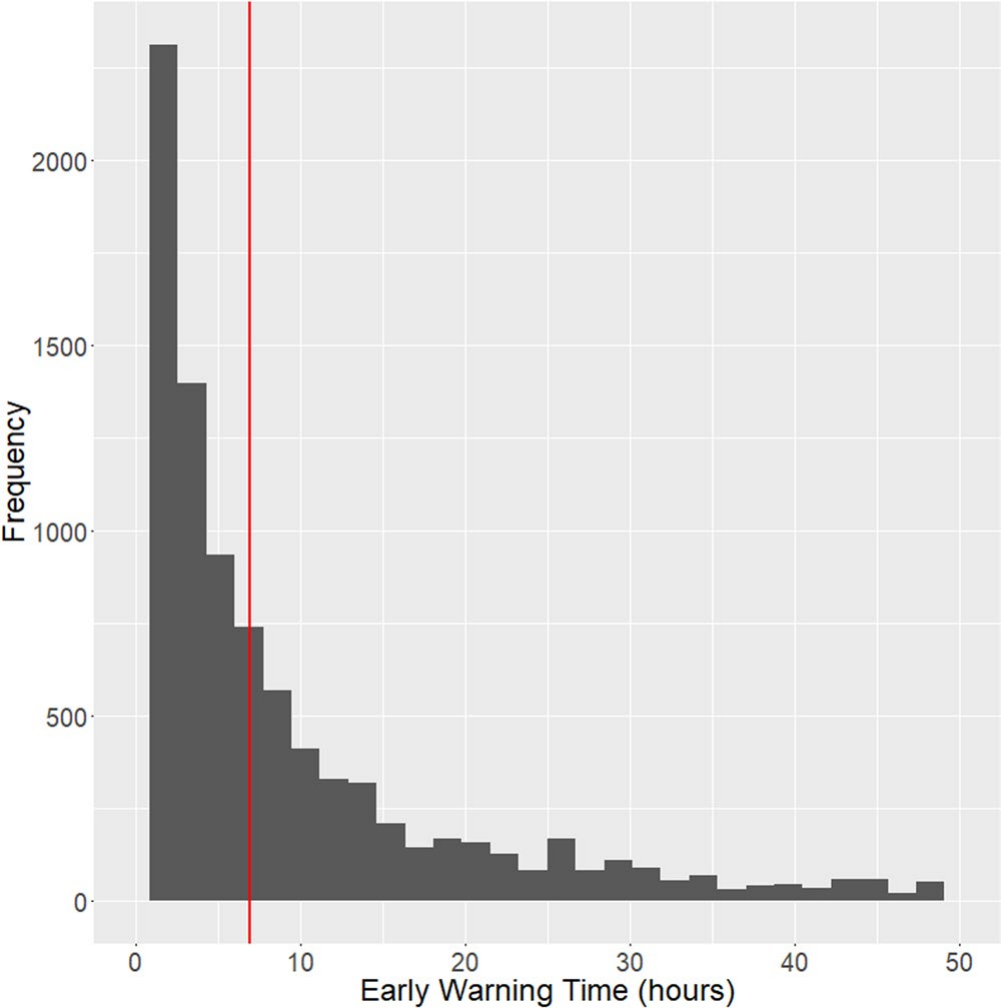
Histogram of EWT over all bootstrapped iterations. Red vertical line indicates median value of 7 hours.

The major performance differences between our pre-shock state-based method vs the Cox method as employed previously by Henry *et al*.^10^ are significantly improved AUC using all 3 machine learning methods, significantly improved sensitivity and EWT using GLM and RNN, and significantly improved PPV using RNN. Out of the three machine learning methods used, RNN achieves the highest performance across all criteria, achieving 0.93 AUC, 88% sensitivity, 84% specificity, an overall 52% PPV, and a median EWT across all true positive cases of 7.0 hours (IQR 2.5 to 22.8 hours). The distribution of EWT for this method is shown in Fig. 5. Using the Wilcoxon rank-sum^19^ test, our RNN-based method outperforms the Cox proportional hazards model in AUC (p = 1.85e-10), sensitivity (p = 3.06e-7), PPV (p = 0.0010), and EWT (p = 4.65e-11) with 99% confidence. Compared to our GLM-based method, the second highest-performing method, our RNN-based method achieves significantly higher AUC (p = 5.45e-9) and sensitivity (p = 7.57e-7).

**Figure 6:**
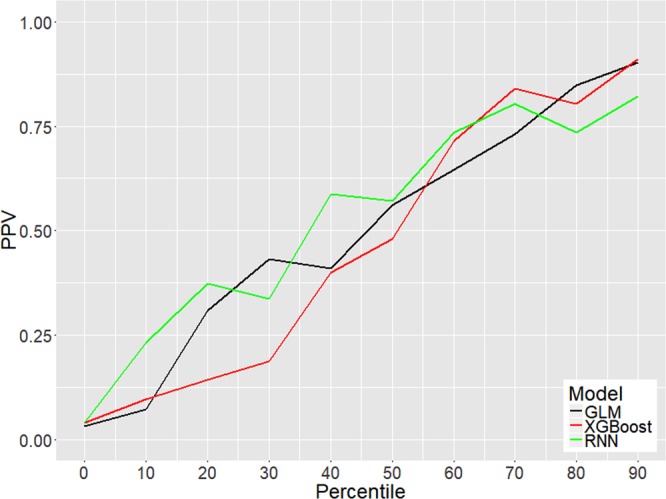
Positive predictive value shown as a function of each decile in the distribution of *z*_θ_.

Our approach allows for calculation of a patient-specific PPV. This was done using the first updated value of the risk score that exceeds the threshold (*z*_θ_). The range of *z*_θ_ values were separated into deciles, and PPV was computed for each decile yielding an estimate of the probability that each prediction is a true positive. Figure 6 shows PPV for each decile. For all three methods, in higher deciles, the likelihood that the prediction is a true positive is higher than in lower deciles; PPV in the top 10% ranges between 0.82 (for RNN) and 0.91 (for XGBoost).

EWT is also a monotonically increasing function of dataset length, which is defined as the time interval between the first measured data point and the time of septic shock onset (see Supplement: *Impact of Dataset Length on EWT*). This finding suggests that continuous data collection and analysis during and even before an ICU stay will help increase EWT.

The pre-shock state is physiologically distinct from both the sepsis state and the state of septic shock itself. Table 2 shows that the values of the top 3 features from Fig. 3 exhibit significant changes (Wilcoxon signed-rank^19^) upon transition from sepsis to pre-shock, and from pre-shock to septic shock in a group of patients who all progress from sepsis to septic shock. Heart rate does not change significantly over time but is elevated relative to the population baseline. Fraction of inspired oxygen (FiO_2_) does not change over time; however, the presence of FiO_2_ values indicates that the patient is being mechanically ventilated. Partial pressure of oxygen (PaO_2_) increases slightly between sepsis and pre-shock, then decreases significantly with septic shock onset (see Supplement, *Characterization of the Pre-Shock State* for further discussion). At the time of our early warnings, 62% of septic shock patients have not received any form of vasopressor treatment, 81% have not received adequate fluid resuscitation, and 56% have received neither vasopressors nor adequate fluid resuscitation. Transition into the pre-shock state therefore has clinical significance since it indicates a high likelihood of future transition to septic shock, and detection of this transition occurs at a time when therapeutic interventions remain an option.

**Table 2:** Evolution of patient physiology during progression from sepsis to pre-shock to septic shock for top six physiological features.

Physiological Feature	Sepsis	Pre-shock	Septic shock
Lactate (mmol/L)	3.15 ± 2.71	4.57 ± 3.69*	4.98 ± 3.67*
Cardiovascular SOFA	0.55 ± 0.90	1.72 ± 0.70*	1.97 ± 0.24*
GCS	9.72 ± 4.26	8.22 ± 4.01*	7.72 ± 3.80^†^
HR (bpm)	97.7 ± 22.2	97.3 ± 22.4	96.2 ± 20.3
PaO_2_ (mmHg)	127.4 ± 71.1	132.4 ± 78.2	119.1 ± 68.7*
FiO_2_	0.62 ± 0.24	0.61 ± 0.22	0.59 ± 0.20

Values are given as mean ± standard deviation. An asterisk (*) indicates that the change in value from the preceding state is statistically significant with 99% confidence (p < 0.01). A dagger (†) indicates that the change in value from the preceding state is statistically significant with 95% confidence (p < 0.05).

## Discussion

### Prediction method

The role of automated methods for early prediction of sepsis and septic shock is to alert clinicians when the underlying physiology is indicative of increasing possibility of deterioration of patient state, thus providing a greater window of time for intervention. In this work, we hypothesize that it is possible to identify the presence of a physiological signature in a sepsis patient that defines what we refer to as the “pre-shock” state. Transition into the pre-shock state increases the likelihood of the patient’s impending transition into septic shock. We use this signature to calculate a risk score. When that risk score exceeds a fixed threshold, we predict that a transition to septic shock will occur. Results show that this method yields increased performance over a Cox proportional hazards model-based method. Perhaps the most important performance metric achieved is an overall positive predictive value of 52% with PPV in the highest decile reaching as high as 91%. Minimizing the frequency of false alerts (i.e. achieving high PPV) is essential for the usability of an algorithm in the clinical setting. Early prediction of septic shock is achieved with a median EWT of 7 hours. This increased EWT enables earlier interventions, such as the consensus recommendations of the 2016 Surviving Sepsis Campaign^20^.

### Patient-specific positive predictive value

We introduce the notion of patient-specific positive predictive value. By splitting patients into cohorts according to the first value of risk score immediately following threshold crossing *z*_θ_, it is possible to stratify the likelihood that a positive prediction is a true positive. This allows our detection method to estimate the confidence with which a positive prediction is made and provides more reliable and actionable information to clinicians than would an alert alone. For some patients this confidence is as high as 75–90%. In clinical practice, physicians may choose to act only on high-confidence predictions or may elect to take different courses of action depending on the certainty with which imminent transition into septic shock is predicted.

### Interpretation of model

While RNNs yield the greatest performance for early prediction of septic shock, GLM is the most interpretable of the three models built, and thus we rely upon results obtained using GLM for interpretation of the model. The pre-shock state was defined using a data-driven machine learning approach. The magnitude of the exponentiated GLM coefficients presented in Fig. 3 can be interpreted as the relative contribution of each feature to the risk score. Based on the relative magnitude of the weights for each normalized feature, lactate, cardiovascular SOFA score, heart rate, PaO_2_, FiO_2_, and GCS are the six most important indicators that a sepsis patient is at elevated risk of entering septic shock. These findings are in line with existing literature on the pathophysiology of sepsis and septic shock: elevated serum lactate is an indicator of reduced tissue perfusion, is associated with increased mortality in patients with infections, and has been previously suggested as a biomarker for sepsis patient risk stratification^21–23^. Increased cardiovascular SOFA score is in turn indicative of more severe cardiovascular dysfunction. Decreased GCS and altered mental status in sepsis patients has similarly been associated with increased mortality^24,25^. The presence of FiO_2_ values in the EHR are indicative of mechanical ventilation; elevated PaO_2_ is also indicated by our model as a factor contributing elevated risk of transition into septic shock, and has been linked to greater mortality^26^_._ However, because of a lack of complete data in the MIMIC-III clinical database on ventilator usage, it is not possible to precisely verify a link between ventilator usage, hyperoxemia, and increased severity of patient state in the study population.

### Clinical state labels

The use of supervised learning techniques in early prediction of clinical state transition necessitates accurate determination of clinical state labels. Under the Sepsis-2 definitions, patients with systemic inflammatory response syndrome (SIRS) and suspected infection are considered to have sepsis. Vincent *et al*. point out that the SIRS criteria are extremely sensitive, with up to 90% of ICU patients meeting them at some point during their stay^27^. Here it is demonstrated that clinical state labels generated using Sepsis-2 criteria exhibit substantially more frequent fluctuations over time (i.e. temporal instability) than those using Sepsis-3 criteria. The temporal stability of Sepsis-3 criteria is in large part due to the fact that it relies on SOFA scores which are evaluated based on the most severe physiological values observed over the preceding 24 hours, whereas the SIRS-based Sepsis-2 criteria are subject to change with each new observation. In our view, the analyses described in this study would not have been possible using Sepsis-2 definitions due to the large number of clinical state fluctuations. This fundamental limitation of Sepsis-2 impacts the ability to reliably know the clinical state of a patient at any given moment in time and suggests that all prior work that relied on Sepsis-2 may require reevaluation.

Studies that use the Sepsis-2 definitions determine suspected infection using ICD-9 codes, as specified by Angus *et al*. in 2001^28^. Seymour *et al*.^29^ lay out new criteria for determining suspected infection when evaluating the Sepsis-3 criteria: suspected infection requires that both antibiotics and body fluid cultures be present within a specific time window; time of suspected infection is determined as the earlier of these two orders. These criteria are utilized in more recent studies such as Nemati *et al*.^11^ and Shashikumar *et al*.^30^ We find that this change in criteria for determining suspected infection has implications on the resulting clinical state labels. When Sepsis-3 criteria are computed using different criteria for suspected infection, the set of patients diagnosed with sepsis changes by up to 30% depending on which suspected infection criteria are used (see Supplement, *Infection Criteria*). Nonetheless, our prediction strategy is still able to reliably identify those patients with sepsis who transition to septic shock.

### Limitations

The clinical state transition being studied is defined by the clinical state labels, and as a result, any limitation of the labels themselves are also limitations of the study. Outside of the highly-monitored environment of the ICU, there may not be sufficient data to evaluate the Sepsis-3 criteria. In other settings such as the emergency room, it may be necessary to use different definitions of sepsis such as qSOFA^1^ or SIRS^18^. Further study using these definitions may be merited.

The MIMIC-III database from which the data for this study were drawn is comprised of ICU patients from a single hospital. Attempting to use the same model parameters learned in this study outside of this setting may yield decreased performance, as patient physiology may vary from population from population. However, we show as part of this study that moderate performance using our models trained on MIMIC-III can be achieved on a the Phillips/MIT eICU^31^ multi-center database (0.82/0.83/0.85 AUC with GLM/XGBoost/RNN respectively), demonstrating that our method is sufficiently robust that models trained on a single-center database can be applied to data from multiple hospitals (see Supplement, *Validation in an Independent Cohort*). In practice, deploying automated early prediction methods should involve fitting a new model specific to the population of interest after sufficient data has been collected, as training a model on a data set that is representative of the new setting will result in better performance than simply employing the original model.

### Outlook

We show that by characterizing the pre-shock state using a variety of machine learning methods, it is possible to achieve early prediction of impending transition to septic shock. Best performance is obtained using recurrent neural networks, achieving early prediction of septic shock with an AUC of 0.93, 88% sensitivity, 84% specificity, 52% overall positive predictive value, and a median EWT of 7 hours, which provides an actionable window of time for therapeutic intervention. Furthermore, we show that patient-specific PPV, which can be as high as 75–90%, provides an estimate of the confidence when a positive prediction is made and provides more reliable and actionable information to clinicians than would an alert alone. The paradigm in which a clinical state such as sepsis is subdivided into distinct, temporally adjacent states, the latter of which (pre-shock) is a transitional state that is only occupied if the patient is highly likely to progress along the clinical spectrum to a subsequent clinical state (septic shock), can potentially be applied to many other clinical state transition prediction problems in ICU patients. We find also that the Sepsis-3 criteria yield clinical state labels which are temporally stable, whereas the SIRS-based Sepsis-2 criteria yield labels which fluctuate frequently over time, reducing its usefulness for defining clinical state labels for predictive algorithms.

## Methods

### Data extraction and processing

The version 1.4 MIMIC-III clinical database contains data from 38,645 adult patients admitted to the ICUs of Beth Israel Deaconess Medical Center from 2001 to 2012^17^. Of these, 15,930 patients had suspected infection, as determined by concomitant orders for antibiotics and blood cultures. EHR data for these patients was extracted from the MIMIC-III PostgreSQL database using the RPostgreSQL package^32^. The majority of data entries in MIMIC-III are comprised of timestamp-value pairs with a numeric subject id, identifying which subject the entry belongs to, and a numeric itemid identifying the meaning of the value of the entry. A complete table of all itemids corresponding to each physiological variable used in this study, along with further information on processing and interpretation of data for individual features can be found in the Supplement (Table S1, *Data Extraction and Processing*). 70% of patients were randomly sampled into the training set, whereas the remaining 30% were reserved for testing, which we refer to as the testing set.

### Labeling clinical states

The Third International Consensus Definitions for Sepsis and Septic Shock (Sepsis-3)^1,33^ were applied to determine patient state (non-sepsis, sepsis, or septic shock) at each measurement time point. Sepsis patients are those with suspected infection and a Sequential Organ Failure Assessment (SOFA) score of 2 or higher^1,15^. Septic shock patients are those who have sepsis, have received adequate fluid resuscitation, require vasopressors to maintain a mean arterial blood pressure of at least 65 mmHg, and have a serum lactate >2 mmol/L^33^. Time of septic shock onset (t_o_) is determined as the earliest time when all the conditions of septic shock are satisfied.

### Risk modeling

Figure 1A shows the risk score (black line) of a patient who transitions from sepsis to septic shock; we define the time of this transition as onset time (t_o_). We assume that in patients who transition from sepsis to septic shock, there exists a clinical state of sepsis that we refer to as the “pre-shock” state. Three different machine learning methods were used to characterize the pre-shock state: GLM, XGBoost, and Recurrent Neural Networks (see Supplement: *Recurrent Neural Networks* for additional information on training the RNN model and choosing network structure). For the chosen machine learning method, a regression model is trained using data from the sepsis clinical state in patients who did not go into septic shock, and data from a 1-hour time window prior to septic shock onset in patients who do transition to septic shock. Because the exact duration of the pre-shock state is unknown, models were trained using data from different hour-long windows beginning at 12 hours preceding septic shock onset to 3 hours after septic shock onset. The window spanning from 2 hours prior to septic shock onset to 1 hour prior to septic shock onset achieved the best detection performance and was selected. Note that data immediately following the time of transition into septic shock were not used in model training because according to the Sepsis-3 definition of septic shock, patients are being treated with vasopressors at this time.

We also implemented the Cox proportional hazard modeling approach described recently by Henry *et al*.^10^ and compared results using that risk model. Lasso regularization was used to select features that yielded best performance for both GLM and Cox; model selection was conducted using 10-fold cross validation on the training set.

### Prediction

For early detection of septic shock, each patient’s risk score is calculated for each unique timestamp for which there is EHR data from the beginning of their observations until septic shock onset. Detection occurs at the first time at which a patient’s risk score exceeds the threshold value. We define the time of this first observation of risk exceeding the threshold as the detection time. The optimal detection threshold is determined from the ROC curve as the value of the threshold corresponding to the point on the ROC curve closest to the upper left-hand corner (closest to the point true positive rate = 1, false positive rate = 0). We define early warning time (EWT) as the difference between onset time and detection time.

### Evaluating model performance

All hyperparameter optimization and model selection was conducted using the training set. Performance criteria are then evaluated using these models and thresholds on the testing set. Statistical tests for comparison of performance metrics between different modeling approaches were conducted on sampled values of performance metrics obtained using bootstrap.

### Image integrity statement

All figures were generated using the ggplot2^34^ package. Multi-panel figures were composited using Adobe Photoshop CC 2019.

## Supplementary information

Supplementary Materials

## Data Availability

Data analyzed in this study are publicly available from the MIMIC-III database (https://mimic.physionet.org), version 1.4. To enhance reproducibility, we will make extracted data used in this analysis available in R workspace image format (.rdata), along with all data analysis methods and executable code for reproducing each figure on the Institute for Computational Medicine website (https://icm.jhu.edu/software/).

## Code Availability

All code used to extract, process, and analyze data will be made available on the Institute for Computational Medicine website (https://icm.jhu.edu/software/). All external R libraries used for data extraction, processing, or analysis are available via CRAN (https://cran.r-project.org/). Analysis using deep learning utilized the TensorFlow back-end (https://www.tensorflow.org/) and the R interface for Keras (https://keras.rstudio.com/).
