# Supplementary material for: Data-driven discovery of a novel sepsis pre-shock state predicts impending septic shock in the ICU
Source: Sci Rep. 2019 Apr 16;9:6145. doi: 10.1038/s41598-019-42637-5 (PMC6467982; doi:10.1038/s41598-019-42637-5)
Supplement: Supplementary file 1 — Supplementary Materials [file 41598_2019_42637_MOESM1_ESM.docx]

**Title: Data-driven discovery of a novel sepsis pre-shock state predicts impending septic shock in the ICU**

**Supplementary Materials**

**Authors:** Ran Liu, BS­^1,2^, Joseph L. Greenstein, PhD^1^, Stephen J. Granite, MS, MBA^1^, James C. Fackler, MD^3^, Melania M. Bembea, MD, MPH, PhD^3,4^, Sridevi V. Sarma, PhD^1,2^* & Raimond L. Winslow, PhD^1,2^*

**Affiliations:**

^1^Institute for Computational Medicine, The Johns Hopkins University

^2^Department of Biomedical Engineering, The Johns Hopkins University School of Medicine & Whiting School of Engineering

^3^Department of Anesthesiology and Critical Care Medicine, and ^4^Department of Pediatrics, The Johns Hopkins University School of Medicine

*To whom correspondence should be addressed: [rwinslow@jhu.edu](mailto:rwinslow@jhu.edu) & [ssarma2@jhu.edu](mailto:ssarma2@jhu.edu)

Note: Authors R.L. Winslow & S.V. Sarma contributed equally.

**Materials and Methods:**

*Data Extraction and Processing*

MIMIC-III contains 38,418 adult patients with at least one entry of the queried features. EHR data for these patients was extracted from the MIMIC-III PostgreSQL database. The majority of data entries in the MIMIC-II database consist of timestamp-value pairs; each entry also has a numeric subject id, identifying which subject the entry belongs to, and a numeric itemid identifying the meaning of the value of the entry. A separate table contains a list of all itemids present in the database, along with an accompanying description of the data contained in all entries with that itemid (e.g. HR, SBP, etc.). Multiple itemids may correspond to the same feature; for those features, all MIMIC-III itemids specified in Table S1 were queried. If there were multiple entries for a feature for a single time stamp, the mean value of the non-empty entries was used. In the case of administration of medication, some items report dosages in varying units of measure. All values were converted to mcg/kg/min. Similarly, temperature was sometimes reported in degrees Celsius, and sometimes in degrees Fahrenheit. All values were converted to degrees Fahrenheit. Fluid administration and urine output were calculated from the MIMIC-III inputevents_cv and inputevents_mv tables, a table containing entries of fluid input and output for patients. Age of each patient was determined by subtracting the date of birth of each patient from the timestamps of their EHR data.

There is a split in MIMIC-III due to the change in EHRs used at Beth-Israel Deaconess Medical Center: data from 2008 and earlier come from CareVue, whereas data from 2008 onward come from MetaVision. Therefore, the format of data in MIMIC-III changes according to the EHR from which patient data originated. Certain steps in data processing must be taken to address these discrepancies in data format. GCS from CareVue is reported as a net score, whereas in MetaVision, each component of GCS is given individually; therefore, MetaVision entries for GCS must be summed for each timestamp. Input events from MetaVision data (in the inputevents_mv) table have a start and end time, whereas CareVue entries only report a single timestamp. The time at which a fluid input or infusion drug administration occurred for MetaVision entries is considered to be equal to the start time.

*Labeling Clinical States*

The definition of adequate fluid resuscitation follows that of the Surviving Sepsis Campaign guidelines for treatment^1,2^, which recommend 30 mL/kg of fluids over three hours, with treatment targets of urine output >0.5 mL/kg/hr and CVP of 8-12 mmHg. Based on this definition, a patient is considered adequately fluid resuscitated if, in the past three hours, they have been administered at least 30 mL/kg of fluids, or if the treatment targets of urine output >0.5 mL/kg/hr or CVP 8-12 mmHg have been met.

*Infection Criteria*

A requirement of both the Sepsis-2 and Sepsis-3 criteria is the identification of patients with suspected infection. All sepsis and septic shock determined under these criteria fulfill criteria for suspected infection. The consensus definitions themselves suggest the usage of concomitant orders for antibiotics and body fluid cultures as indicators of suspected infection; subsequent work using the Sepsis-3 criteria, including studies by Seymour et al., Nemati et al., and Shashikumar et al., apply these criteria ^3-5^.

Earlier work using the Sepsis-2 criteria, such as the Henry et al. study, determine suspected infection using ICD-9 codes specified by Angus et al ^6,7^. The set of patients identified as having suspected infection varies, depending on which criteria for suspected infection are applied. Out of the 38,418 patients with at least one entry of EHR data in the MIMIC-III database, 15,930 have suspected infection according to the presence of concomitant orders for antibiotics and blood cultures, whereas 16,912 have suspected infection according to their ICD-9 codes, and 12,206 have suspected infection according to both criteria. This in turn affects determination of the set of sepsis patients and septic shock patients; if suspected infection is determined according to the presence of concomitant orders for antibiotics and blood cultures, 15,111 patients are labeled as septic, and of these, 3,475 are labeled as in septic shock. If ICD-9 codes are used instead, 15,932 patients are labeled as septic, and of these, 3,507 are labeled as in septic shock. Of all sepsis patients, 11,591 meet both criteria. Of all septic shock patients, 2,892 meet both criteria. Although the majority of patients meet both criteria, the composition of up to 30% of the set of sepsis patients changes depending on which criteria for suspected infection are applied.

*Recurrent Neural Network Structure*

Our selected current neural network structure consists of 3 hidden GRU layers with rectified linear (ReLU) activation. The recurrent neural network consisted of 3 hidden LSTM layers (relu activation function) with 16 units each, and one dense layer, as illustrated in Figure S1. Input consisted of 10 features evaluated at the current time, as well as t-1, … t-12. Prior to training, the training data was rebalanced by upsampling the preshock training instances. 64 epochs, and a batch size of 512. The best model by validation loss was selected from the models generated at the end of each epoch. The loss function was given by binary crossentropy, and the optimizer used was rmsprop.

**Discussion**

*Validation in an Independent Cohort*

The eICU database (v2.0)^8^, released in 2018 contains data from over 200 hospitals, spanning ~200,000 ICU stays, and ~140,000 unique patients. Using this additional data as an independent cohort for validation, i.e. training on MIMIC-III, testing on eICU, we validate our model. As there is limited data in the eICU database on blood cultures, infection criteria were evaluated using ICD-9 codes. Moderate performance is achieved using all three of models trained on MIMIC-III, a single-center database, and applying them to eICU, a multi-center database.

|  | **GLM** | **XGBoost** | **RNN** |
| --- | --- | --- | --- |
| AUC | 0.82 | 0.83 | 0.85 |
| Sensitivity | 0.85 | 0.77 | 0.79 |
| Specificity | 0.73 | 0.73 | 0.77 |
| PPV | 0.23 | 0.22 | 0.26 |
| Median EWT | 9.5 hours | 9.0 hours | 10.3 hours |

*Characterization of the Pre-Shock State*

Twelve hours or more prior to detection, the physiology of sepsis patients who develop septic shock is similar to that of sepsis patients who never enter septic shock (Table S12). There are detectable moderate differences in each variable, with the largest differences occurring in lactate, and heart rate. This observation suggests that changes in different physiological variables occur on different time scales, and that these features may be the leading indicators for early progression toward the pre-shock state.

If we use GLM to classify individual data points from sepsis patients who do not go into septic shock from data in the pre-shock sepsis state, we can do so with 0.94 AUC. Similarly, if we do this with data from pre-shock and data from the septic shock state, we can do so with 0.89 AUC. This indicates that the pre-shock state is physiologically distinct from both sepsis and septic shock.

*Impact of Dataset Length on EWT*

Henry et al. reported a median EWT of 28 hours when using the SIRS-based Sepsis-2 criteria^7^. We attempted to reproduce this finding by generating clinical state labels using the same Sepsis-2 clinical criteria employed in Henry et al. rather than the Sepsis-3 criteria used in this study. However, the temporal instability (see Figure 2) of Sepsis-2 clinical labels makes it difficult to reliably identify when a patient is in septic shock. The time interval between the first measured data point and time of septic shock onset (referred to as “dataset length”) is an upper bound on EWT. Median dataset length also sets the upper bound on median EWT. When Sepsis-3 diagnostic criteria are used, median dataset length and thus the maximum possible median EWT is 12.9 hours. To further illustrate the effect of dataset length on EWT (Fig. S2), analyses were repeated while excluding datasets shorter than a given minimum length. As minimum dataset length increases, median EWT increases from 6.9 to 38.7 hours as shorter datasets are excluded. In addition, ~30% of the true positive detections occur in the first minute of patient observations, indicating that patients have already entered the pre-shock state at the time of ICU admission. In these cases, had data been available from earlier times, the EWT achieved would have been greater. These findings point out that continuous collection and analysis of patient EHR and PTS data is necessary to achieve the maximum EWT.

**Supplementary Figures:**

**
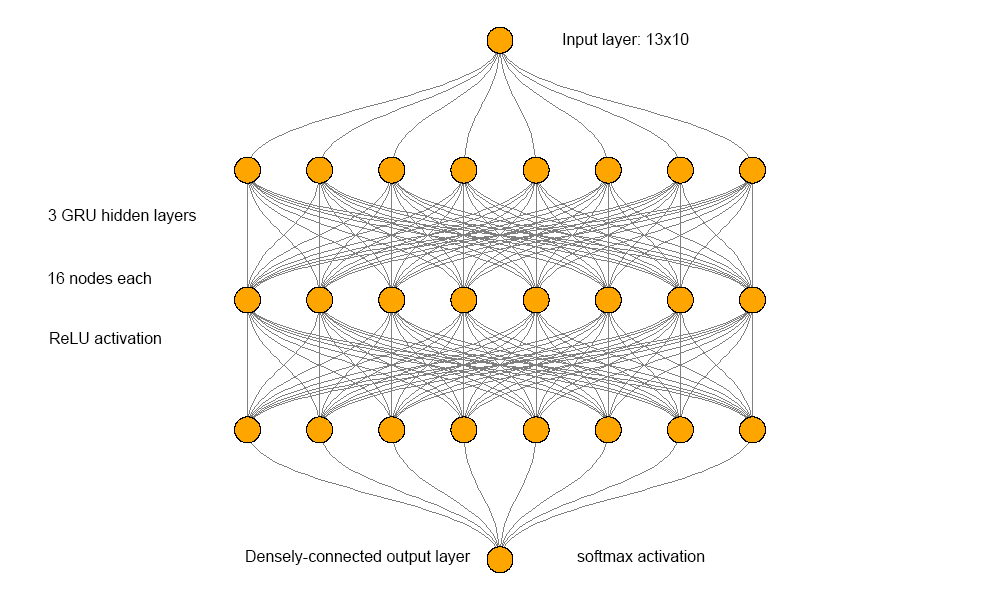
**

**Figure S1:** Recurrent neural network structure diagram. Our neural network consists of 3 hidden layers with 16 GRU units each with ReLU activation, then a single densely-connected layer with softmax activation.


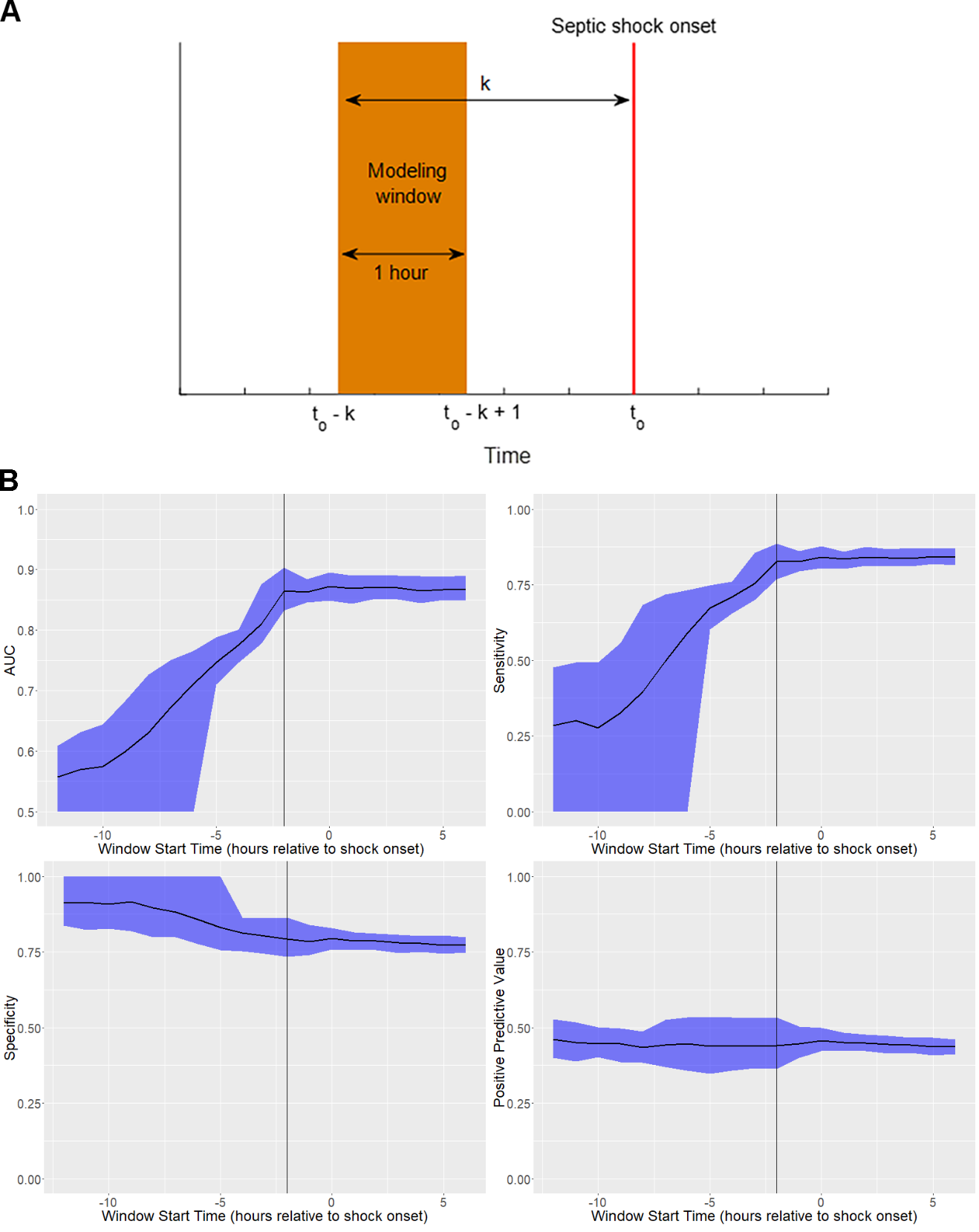


**Figure S2:** Performance for 1-hour sliding windows with 95% confidence intervals. Data from (t_o_-k hours to t_o_-k+1 hours) is used to characterize the transitional sub-state; our models (GLM, XGBoost, RNN) are trained using data from sepsis patients who never go into shock, and data from this 1-hour window. Performance for the window used in results (t_o_ – 2 to t_o_ – 1) is denoted by the vertical line.


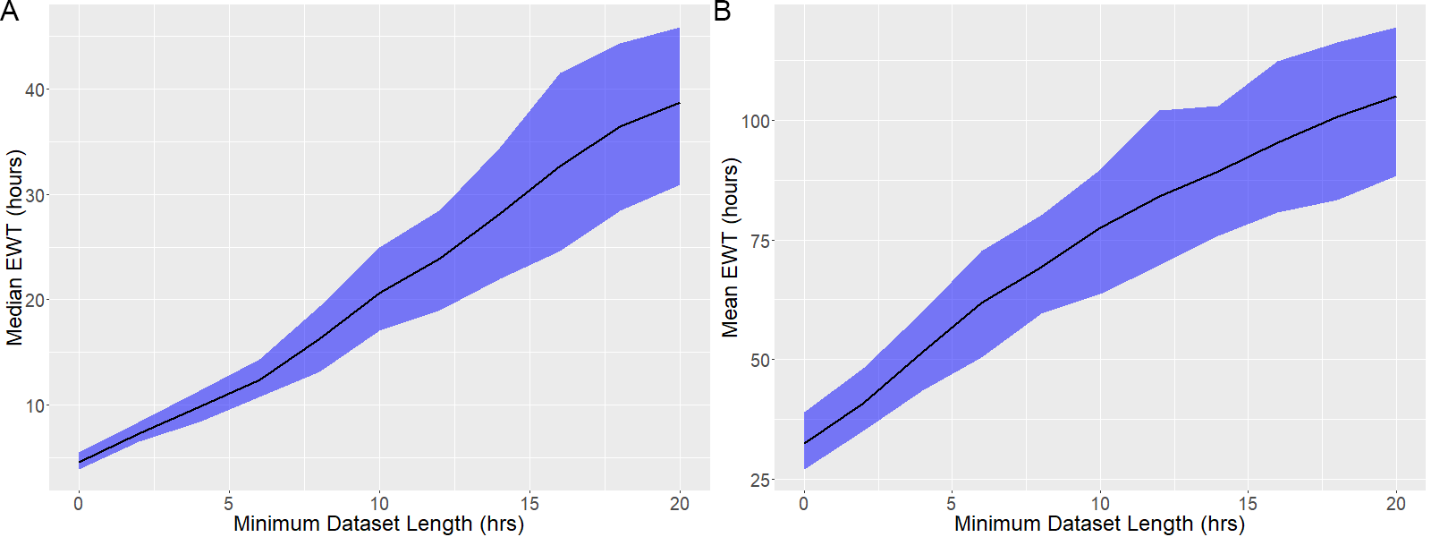


**Figure S3:** Performance vs minimum dataset length. For each value of minimum dataset length, all datasets shorter than the minimum dataset length were excluded from the analysis. Mean values across all bootstrap iterations are indicated by the bold line, and 95% confidence intervals are indicated by the shaded area.


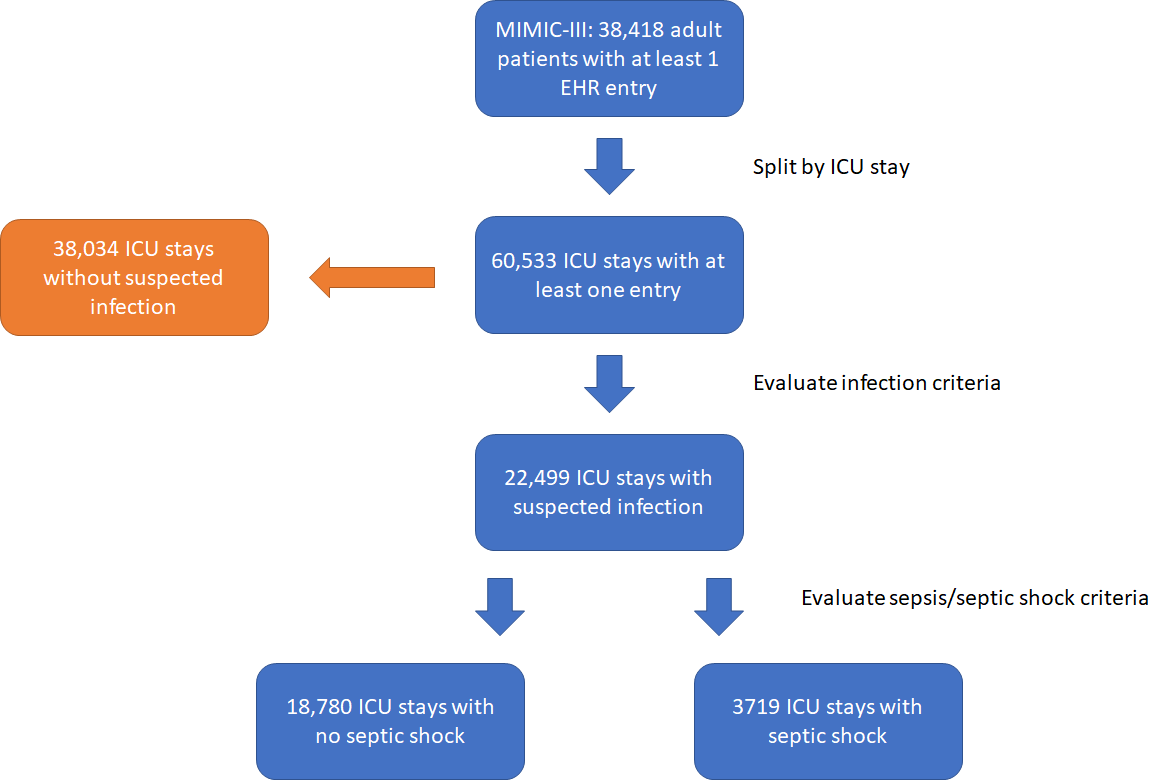


**Figure S4:** Flow diagram illustrating exclusion criteria for data in this study. Orange datasets are excluded from the study, whereas blue datasets are retained at each step. Ultimately, 11,636 sepsis patients and 3,475 septic shock patients are retained and used in the study.


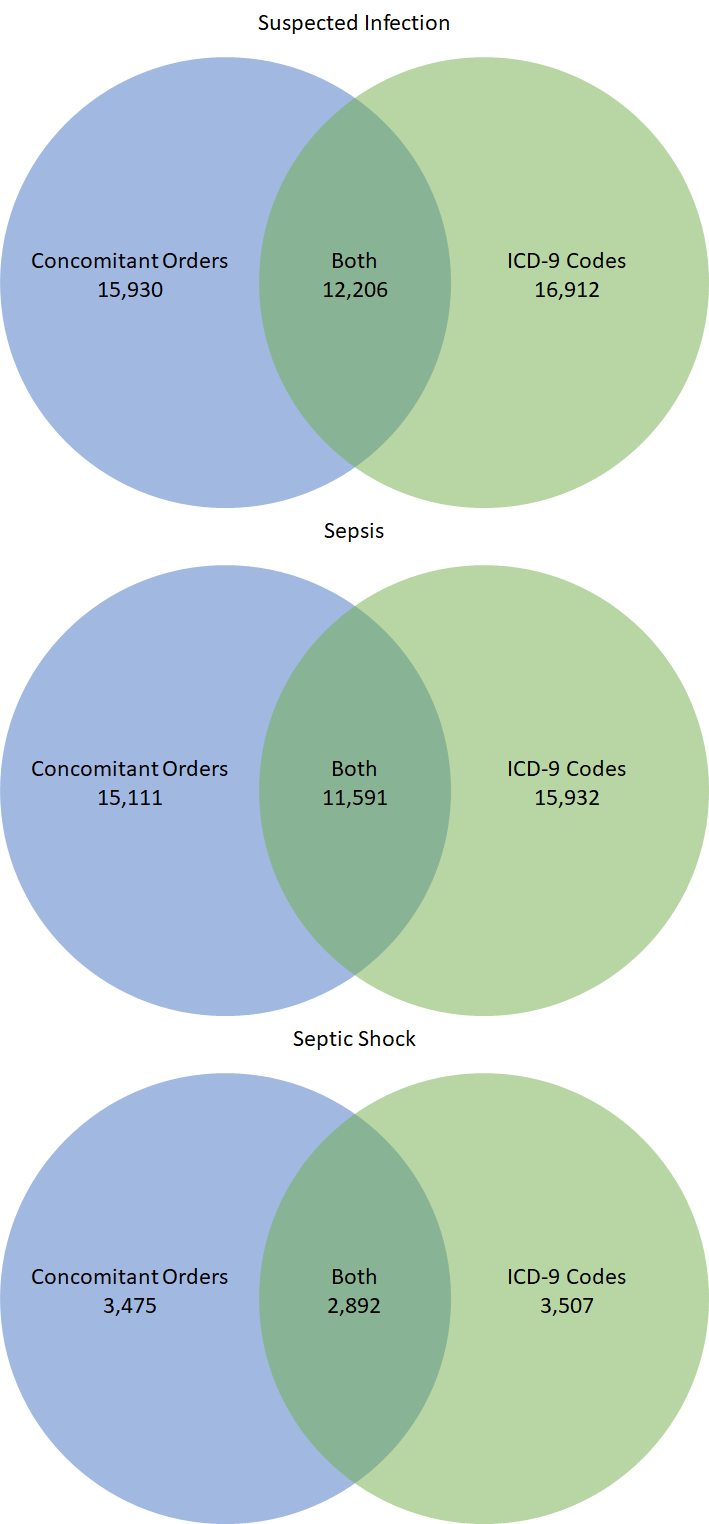


**Figure S5:** Venn Diagram illustrating how applying different infection criteria affects the set of patients identified as having suspected infection, sepsis, and septic shock.

**Supplementary Tables:**

**Table S1: Queried Items in MIMIC-III**

This table contains all the itemid values corresponding to specific clinical variables. Values in the chartevents itemid column were queried from the chartevents table in the MIMIC-III PostgreSQL database. Values in the medevents itemid column were queried from the inputevents_cv, and inputevents_mv table. Urine output was queried from the outputevents table.

| **Feature** | **Chartevents itemid** | **Inputevents itemid** |
| --- | --- | --- |
| Heart rate | 211, 220045 |  |
| Respiratory rate | 615, 618, 8113, 3603, 224690, 220210 |  |
| Temperature | 676, 677, 678, 679, 223761, 223762 |  |
| SBP | 6, 51, 6701, 220050, 225309 |  |
| SBP (noninvasive)* | 442, 455, 3313, 220179 |  |
| DBP | 8364, 8368, 8555, 220051, 225310 |  |
| DBP (noninvasive)* | 8440, 8441, 8502, 220180 |  |
| Mean BP | 52, 6702, 6927, 456 |  |
| Mean BP (noninvasive) | 443, 3312, 220181 |  |
| CVP | 716, 1103, 113, 220074 |  |
| PaO_2_ | 490, 779, 3785, 3837, 220224 |  |
| FiO_2_ | 190, 191, 3420, 3422, 1863, 2518, 2981, 7570, 223835 |  |
| GCS | 198 |  |
| GCS (MV)** | 220739, 223900, 223901 |  |
| Bilirubin | 848, 5483, 5543, 4049, 3220, 5821, 1583, 5032, 5045, 4354, 225690 |  |
| Platelets | 828, 3789, 6256, 227457 |  |
| Creatinine | 791, 3750, 1525, 220615 |  |
| Lactate | 818, 1531, 225668 |  |
| BUN | 1162, 781, 5876, 3737, 225624 |  |
| Arterial pH | 1126, 4753, 780, 223830 |  |
| WBC | 1127, 861, 4200, 1542, 220546 |  |
| PaCO_2_ | 777, 778, 3784, 3835, 220235 |  |
| Hemoglobin | 814, 220228 |  |
| Hematocrit | 813, 3761, 226540 |  |
| Potassium | 829, 3792, 1535, 4194, 227442, 227464 |  |
| Epinephrine |  | 3112, 5752, 30119, 30309, 30044, 221289 |
| Dopamine |  | 4501, 5329, 30043, 30307, 221662 |
| Dobutamine |  | 5747, 30306, 30042, 221653 |
| Norepinephrine |  | 221906, 30047, 30120 |
| Phenylephrine |  | 5656, 6752, 6090, 221749, 30127, 30128 |
| Vasopressin |  | 4501, 5329, 30043, 30307, 221662 |
| Urine output | 40405, 40428, 40534, 41857, 42001, 42362, 42463, 42507, 42510, 42556, 42676, 43171, 43173, 43175, 40288, 42042, 42068, 42111, 42119, 42209, 40715, 40056, 40061, 40085, 40094, 40096, 43897, 43931, 43966, 44080, 44103, 44132, 44237, 44313, 43348, 43355, 43365, 43372, 43373, 43374, 43379, 43380, 43431, 43462, 43522, 44706, 44911, 44925, 42810, 42859, 43093, 44325, 44506, 43856, 45304, 46532, 46578, 46658, 46748, 40651, 40055, 40057, 40065, 40069, 44752, 44824, 44837, 43576, 43589, 43633, 43811, 43812, 46177, 46727, 46804, 43987, 44051, 44253, 44278, 46180, 45804, 45841, 45927, 42592, 42666, 42765, 42892, 43053, 43057, 42130, 41922, 40473, 43333, 43347, 44684, 44834, 43638, 43654, 43519, 43537, 42366, 45991, 227489, 45415, 226627, 226631 | |

*Noninvasive measurements of SBP and DBP are only used if arterial line measurements are unavailable

** GCS in Metavision entries has to be computed as the sum of the individual components

**Table S2: Table of Coefficients**

Model coefficients learned for ten selected features from one sample train/test iteration. Features are listed in descending order of relative importance. Coefficients were learned using data normalized to a mean of 0 and unit standard deviation for each variable.

| **Variable** | **Value** | **SE** |
| --- | --- | --- |
| Intercept | -1.326 | 0.002806 |
| Lactate | 1.690 | 0.006523 |
| Cardiovascular SOFA | 0.5822 | 0.002315 |
| GCS | -0.5602 | 0.003129 |
| HR | 0.3350 | 0.003532 |
| PaO_2_ | 0.2847 | 0.002456 |
| FiO_2_ | 0.2544 | 0.003556 |
| Respiratory Rate | 0.1819 | 0.002753 |
| Kidney SOFA | 0.08121 | 0.002454 |
| Respiratory SOFA | 0.08038 | 0.002559 |
| Coagulatory SOFA | 0.06659 | 0.002529 |

**Table S3: Adjusted Odds-Ratios**

Odds ratios are calculated by exponentiating the coefficients given in Table S2. The lower and upper bounds of the 95% confidence interval for the exponentiated coefficients are given.

| **Variable** | **Exponentiated value** | **95% confidence interval (lower bound)** | **95% confidence interval (upper bound)** |
| --- | --- | --- | --- |
| Intercept | 0.266 | 0.264 | 0.267 |
| Lactate | 5.420 | 5.350 | 5.491 |
| Cardiovascular SOFA | 1.790 | 1.782 | 1.798 |
| GCS | 0.571 | 0.568 | 0.575 |
| HR | 1.398 | 1.388 | 1.408 |
| PaO_2_ | 1.329 | 1.323 | 1.336 |
| FiO_2_ | 1.290 | 1.281 | 1.299 |
| Respiratory Rate | 1.200 | 1.193 | 1.206 |
| Kidney SOFA | 1.085 | 1.079 | 1.090 |
| Respiratory SOFA | 1.084 | 1.078 | 1.089 |
| Coagulatory SOFA | 1.067 | 1.063 | 1.074 |

**Table S4: Statistics and demographic information on MIMIC-III clinical database**

We determine clinical state labels using the Sepsis-3 criteria for each patient in the MIMIC-III database with at least one entry in the EHR for the items queried in Table S1. We assign each patient to one of three cohorts based on the most severe state reached at any point during their data. The prevalence of each cohort and the corresponding in-hospital mortality rates are given. We compute statistics on gender, age, length of ICU stay, and Charlson comorbidity index on these cohorts.

| **Most severe clinical state reached** | **No sepsis** | **Sepsis without shock** | **Sepsis leading to septic shock** |
| --- | --- | --- | --- |
| Number of patients | 23,307 | 11,636 | 3,475 |
| Percentage of all patients | 60.7% | 30.3% | 9.0% |
| In-hospital mortality | 8.8% | 16.7% | 48.1% |
| Gender | 57.3% male, 42.7% female | 55.0% male, 45.0% female | 57.4% male, 42.6% female |
| Mean age in years (SD) | 62.2 (16.8) | 63.2 (16.1) | 65.2 (14.7) |
| Median length of ICU stay in days | 1.3 | 3.1 | 7.4 |
| Mean Charlson comorbidity index (SD) | 2.06 (2.43) | 3.76 (2.71) | 3.81 (2.58) |

**Table S6: Charlson Comorbidities in MIMIC-III:**

For the same cohorts as in Table S5, we compute the prevalence of each individual category of comorbidity contributing to the Charlson comorbidity index.

| **Comorbidity** | **Non-sepsis** | **Sepsis** | **Shock** | **Overall** |
| --- | --- | --- | --- | --- |
| Myocardial infarction | 16.5% | 20.5% | 26.4% | 18.2% |
| Congestive heart failure | 22.8% | 43.3% | 49.8% | 30.2% |
| Peripheral vascular disease | 10.4% | 15.1% | 18.2% | 12.2% |
| Cerebrovascular disease | 12.5% | 17.2% | 12.5% | 13.8% |
| Dementia | 1.3% | 3.6% | 2.4% | 2.1% |
| Chronic pulmonary disease | 19.2% | 34.0% | 28.3% | 24.0% |
| Rheumatic disease | 2.3% | 3.9% | 3.9% | 2.9% |
| Peptic ulcer disease | 2.9% | 5.0% | 4.1% | 3.6% |
| Mild liver disease | 6.9% | 17.6% | 27.5% | 11.2% |
| Diabetes without chronic complication | 2.2% | 36.8% | 37.4% | 27.2% |
| Diabetes with chronic complication | 5.4% | 11.3% | 9.6% | 7.4% |
| Hemiplegia or paraplegia | 2.4% | 4.9% | 3.0% | 3.1% |
| Renal disease | 12.1% | 28.4% | 28.0% | 17.7% |
| Any malignancy, including lymphoma and leukemia, except malignant neoplasm of skin | 9.7% | 16.4% | 15.0% | 11.9% |
| Moderate or severe liver disease | 2.4% | 8.6% | 11.1% | 4.7% |
| Metastatic solid tumor | 5.3% | 7.1% | 7.0% | 5.9% |
| AIDS/HIV | 0.6% | 2.0% | 1.7% | 1.1% |

**Table S7: Availability of EHR Data in MIMIC-III:**

For each physiological variable queried, we compute the average frequency at which it is reported, as the sum across all patients of the durations between the first and last time timestamp for each dataset divided by the number of timestamps with non-empty entries for each feature. This computation is done on datasets split by gaps greater than one week in length. The proportion of datasets with at least one non-empty entry for each feature is given.

| **Feature** | **Mean hours/data point** | **% datasets with at least 1 data point** |
| --- | --- | --- |
| HR | 0.9 | 99.4% |
| Respiratory Rate | 0.9 | 99.4% |
| Temperature | 5.7 | 51.7% |
| Systolic BP | 1.2 | 98.9% |
| Diastolic BP | 1.2 | 98.9% |
| Mean BP | 1.2 | 98.9% |
| CVP | 4.1 | 32.0% |
| PaO_2_ | 16.2 | 53.3% |
| FiO_2_ | 3.9 | 54.3% |
| GCS | 4.6 | 86.5% |
| Bilirubin | 90.9 | 37.7% |
| Platelets | 21.0 | 95.2% |
| Creatinine | 20.2 | 86.7% |
| Lactate | 49.7 | 44.0% |
| BUN | 20.3 | 86.5% |
| Arterial pH | 16.2 | 54.5% |
| WBC | 22.2 | 94.0% |
| PaCO_2_ | 16.2 | 53.4% |
| Hemoglobin | 21.8 | 84.7% |
| Hematocrit | 23.0 | 67.9% |
| Potassium | 12.7 | 90.1% |

**Table S8: Central Tendency Measures of Patient Physiological Data in MIMIC-III:**

For each adult patient in MIMIC-III with at least one entry of the items queried in Table S1, we compute mean and median values for each queried physiological variable. Mean and median are calculated across each non-empty entry in the EHR.

| **Feature** | **Mean Value** | **Median Value** |
| --- | --- | --- |
| HR | 91.2 bpm | 86.0 bpm |
| Respiratory Rate | 20.2 bpm | 20.0 bpm |
| Temperature | 37.0 C | 37.0 C |
| Systolic BP | 121.3 mmHg | 119.0 mmHg |
| Diastolic BP | 61.7 mmHg | 59.0 mmHg |
| Mean BP | 79.3 mmHg | 77.0 |
| CVP | 11.7 | 11.0 |
| PaO_2_ | 133.0 | 110.0 |
| GCS | 9.1 | 7.5 |
| Bilirubin | 4.1 | 1.2 |
| Platelets | 229.1 | 184.0 |
| Creatinine | 1.8 | 1.2 |
| Lactate | 2.8 | 1.8 |
| BUN | 49.2 | 29.0 |
| Arterial pH | 7.4 | 7.4 |
| WBC | 12.5 | 10.9 |
| PaCO_2_ | 37.9 | 36.5 |
| Hemoglobin | 10.0 | 9.8 |
| Hematocrit | 29.9 | 29.7 |
| Potassium | 4.1 | 4.0 |

**Table S9: Most common causes of infection based on ICD-9 codes specified by Angus et al. in MIMIC-III:**

For each adult patient in MIMIC-III with at least one entry of the items queried in Table S1, we determined suspected infection using the ICD-9 codes specified by Angus et al ^6^. The prevalence of the categories of infection in Angus et al. most common in the MIMIC-III database is given here. Note that the categories specified are fairly narrow; pneumonia is spanned by several categories, and that these categories may be overlapping.

| **Cause of infection – ICD-9 code** | **Prevalence** |
| --- | --- |
| Urinary tract infection not otherwise specified – 599.0 | 13.0% |
| Septicemia - 038 | 12.4% |
| Pneumonia, organism not otherwise specified – 486 | 10.0% |
| Bacterial infection in other diseases not otherwise specified - 041 | 9.0% |
| Other bacteria pneumonia – 482 | 4.6% |
| Other cellulitis or abscess – 682 | 3.5% |
| Candidiasis – 112 | 3.4% |
| Intestinal infection not otherwise classified - 008 | 3.3% |
| Bacteremia – 790.7 | 3.2% |
| Peritonitis – 567 | 2.2% |

**Table S10: Provenance of data (determined from records of ICU stays):**

In the icustays table, the location of each ICU stay is given. We report in this table the frequency of each ICU stay location.

| **ICU Stay Location** | **MIMIC-III** |
| --- | --- |
| MICU | 39.4% |
| CCU | 14.5% |
| TSICU | 12.0% |
| SICU | 16.7% |
| CSRU | 17.5% |

**Table S11: Results on different infection criteria**

Early detection performance using our pre-shock state-based method and the Cox proportional hazards model was assessed using two criteria for suspected infection. Clinical state labels were determined using the Sepsis-3 criteria; however, suspected infection was determined using the criterion listed in each column: using ICD-9 codes specified in Angus et al., and using the presence of concomitant orders for antibiotics and body fluid cultures.

| **Method** | **Performance** | **Concomitant Orders** | **ICD-9** |
| --- | --- | --- | --- |
| GLM | AUC | 0.87 | 0.86 |
|  | Sensitivity | 0.82 | 0.81 |
|  | Specificity | 0.83 | 0.81 |
|  | PPV | 0.49 | 0.41 |
|  | Median EWT | 6.9 hours | 6.2 hours |
| XGBoost | AUC | 0.85 | 0.88 |
|  | Sensitivity | 0.76 | 0.77 |
|  | Specificity | 0.79 | 0.88 |
|  | PPV | 0.43 | 0.50 |
|  | Median EWT | 6.0 hours | 5.7 hours |
| RNN | AUC | 0.93 | 0.92 |
|  | Sensitivity | 0.88 | 0.85 |
|  | Specificity | 0.84 | 0.84 |
|  | PPV | 0.52 | 0.46 |
|  | Median EWT | 7.0 hours | 6.2 hours |
| Cox | AUC | 0.82 | 0.83 |
|  | Sensitivity | 0.76 | 0.77 |
|  | Specificity | 0.82 | 0.83 |
|  | PPV | 0.47 | 0.41 |
|  | Median EWT | 6.1 hours | 5.5 hours |

**Table S12: Characterization of Sepsis in Non-Shock and Shock Patients**

Distribution of the six most important physiological features, as determined using coefficients of the GLM is given for the sepsis clinical state in patients who never enter septic shock, and in sepsis at least 12 hours prior to detection in patients who ultimately develop septic shock.

| **Physiological Feature** | **Sepsis in Non-shock Patients** | **Sepsis in Shock Patients** |
| --- | --- | --- |
| Lactate (mmol/L) | 1.83±1.54 | 3.15±2.71 |
| Cardiovascular SOFA | 0.33±0.74 | 0.55±0.90 |
| GCS | 10.40±3.83 | 9.72±4.26 |
| HR (bpm) | 87.7±55.1 | 97.7±22.2 |
| PaO_2_ (mmHg) | 126.5±67.0 | 127.4±71.1 |
| FiO_2_ | 0.49±0.26 | 0.62±0.24 |

1 Dellinger, R. P. *et al.* Surviving Sepsis Campaign: International Guidelines for Management of Severe Sepsis and Septic Shock, 2012. *Intensive Care Medicine* **39**, 165-228, doi:10.1007/s00134-012-2769-8 (2013).

2 Dellinger, R. P. *et al.* Surviving Sepsis Campaign: International guidelines for management of severe sepsis and septic shock: 2008. *Intensive Care Medicine* **34**, 17-60, doi:10.1007/s00134-007-0934-2 (2007).

3 Seymour, C. W. *et al.* Assessment of Clinical Criteria for Sepsis. *Jama* **315**, 762, doi:10.1001/jama.2016.0288 (2016).

4 Nemati, S. *et al.* An Interpretable Machine Learning Model for Accurate Prediction of Sepsis in the ICU. *Critical Care Medicine* **46**, 547-553, doi:10.1097/ccm.0000000000002936 (2018).

5 Shashikumar, S. P. *et al.* Early sepsis detection in critical care patients using multiscale blood pressure and heart rate dynamics. *Journal of Electrocardiology* **50**, 739-743, doi:10.1016/j.jelectrocard.2017.08.013 (2017).

6 Angus, D. C. *et al.* Epidemiology of severe sepsis in the United States: analysis of incidence, outcome, and associated costs of care. *Crit Care Med* **29**, 1303-1310 (2001).

7 Henry, K. E., Hager, D. N., Pronovost, P. J. & Saria, S. A targeted real-time early warning score (TREWScore) for septic shock. *Science Translational Medicine* **7**, 299ra122-299ra122, doi:10.1126/scitranslmed.aab3719 (2015).

8 Pollard, T. J. *et al.* The eICU Collaborative Research Database, a freely available multi-center database for critical care research. *Scientific Data* **5**, 180178, doi:10.1038/sdata.2018.178 (2018).
